# HOXA11-OS participates in lupus nephritis by targeting miR-124-3p mediating Cyr61 to regulate podocyte autophagy

**DOI:** 10.1186/s10020-022-00570-w

**Published:** 2022-11-22

**Authors:** Xiuhong Pan, Shanshan Chen, Ruiwen Shen, Sen Liu, Yanwu You

**Affiliations:** 1 Department of Nephrology, Affiliated Hospital of Youjiang Medical University for Nationalities, No.18 Zhongshan Road II, Baise, 533000 Guangxi Zhuang Autonomous Region China; 2 Department of Nephrology, People’s Hospital of Guangxi Zhuang Autonomous Region, No. 6 Taoyuan Road, Qingxiu District, Nanning, 530000 China

**Keywords:** HOXA11-OS, Autophagy, Lupus nephritis, Cyr61, miR-124-3p

## Abstract

**Background:**

The long chain non-coding RNA HOXA11-OS was recently identified. Increasing studies have shown that HOXA11-OS has regulatory effects on genes in gastric cancer, prostate cancer, and various kidney diseases, but research on its role in systemic lupus erythematosus is still lacking. The present study aimed to investigate the role of HOXA11-OS in the regulation of podocyte autophagy in the development of lupus nephritis (LN) and its potential molecular mechanism.

**Methods:**

mRNA and protein expression of the target gene (i.e., Cyr61) was detected by quantitative real-time polymerase chain reaction, western blotting, and immunofluorescence. Mouse podocytes were induced using serum immunoglobulin G (IgG) from patients with lupus and their viability was detected using the cell counting kit-8 assay. The interaction of miR-124-3p with HOXA11-OS and Cyr61 was analyzed by double luciferase reporter gene assay. Serum autoantibody levels were detected by enzyme-linked immunosorbent assay. Pathological lesions in the kidney tissue were detected by hematoxylin–eosin and periodate-Schiff staining. The independent samples *t*-test was used for comparing two groups, and one-way analysis of variance for comparing multiple groups.

**Results:**

HOXA11-OS was highly expressed in LN tissues, serum, and cells, and the expression of some key autophagy factors and Cyr61 was significantly increased, while miR-124-3p expression was significantly decreased. In vitro, LN-IgG inhibited podocyte activity, increased autophagy and Cyr61 expression, and aggravated podocyte injury in a time- and dose-dependent manner. As a competitive endogenous RNA of miR-124-3p, HOXA11-OS promoted the expression of Cyr61, thus enhancing the autophagy increase induced by LN-IgG and aggravating podocyte injury. Knockdown of HOXA11-OS had the opposite effect. miR-124-3p mimic or Cyr61 knockdown restored the high expression of autophagy factors and Cyr61 induced by HOXA11-OS overexpression and alleviated podocyte injury. Further in vivo experiments showed that injection of sh-HOXA11-OS adeno-associated virus downregulated HOXA11-OS and significantly alleviated renal damage in lupus mice.

**Conclusions:**

HOXA11-OS is involved in the occurrence and development of LN by regulating podocyte autophagy through miR-124-3p/Cyr61 sponging, which may provide a good potential therapeutic target for LN.

## Background

Lupus nephritis (LN) is a kind of glomerulonephritis and the most serious organ injury in systemic lupus erythematosus (SLE) (Narváez 2020). Podocytes are highly differentiated epithelial cells in the kidney that play a key role in maintaining the selective filtration of glomeruli (Nishad et al. 2021; Sakhi et al. 2019). The podocyte marker proteins nephrin and podocin can form the hiatus septum complex, connect foot processes to form the hiatus septum, and prevent proteins and solutes from entering urine from the circulation (Tian et al. 2020; Kravets and Mallipattu 2020). Therefore, maintaining podocyte activity and structural integrity is very important for the clinical treatment of glomerular diseases and chronic kidney diseases.

The opposite strand of homeobox A11 (HOXA11-OS) is a common long non-coding RNA (lncRNA), which can compete with specific microRNAs (miRNAs) as a competitive endogenous RNA (ceRNA), thus changing the expression of downstream key genes and participating in the occurrence and development of many diseases (Wei et al. 2020; Lu et al. 2018a; Zhao et al. 2020). For example, HOXA11-OS can increase the expression of integrin β3 through sponging the miRNA miR-124-3p, which can promote the migration and invasion of gastric cancer (You et al. 2021). However, the mechanism of HOXA11-OS in SLE is still unknown. Therefore, we hypothesize that HOXA11-OS can target miR-124-3p regulatory factors to play a role in LN.

Cysteine rich 61 (Cyr61) is a recently identified secreting stromal cell protein existing at a low level under steady state conditions, which can mediate various cell activities, including cell survival, proliferation, differentiation, migration, adhesion, and synthesis of extracellular matrix (Liu et al. 2019a). It has been confirmed (Lin et al. 2015) that serum Cyr61 level is recognizably increased in SLE patients, which might be due to the increase in Cyr61 expressed in epithelial cells and released into blood through renal blood circulation. In addition, Cyr61 is closely related to clinical disease activity and inflammation of SLE. A recent study (Guo et al. 2021) has shown that during the inflammatory state Cyr61 is involved in the autophagy and apoptosis of renal tubular dermal cells. Autophagy is an intracellular degradation system that maintains cell homeostasis and integrity (Ichimiya et al. 2020). Programmed cell death-1 (Beclin-1) and microtubule associated protein 1 light chain 3 (LC3) are well-known and the most commonly used autophagy-related markers in the autophagy pathway (Vishnupriya et al. 2020; Koustas et al. 2019). In LN, podocytes usually show abnormally high levels of structural autophagy (Zhou et al. 2019; Jin et al. 2019). Therefore, autophagy might be involved in the complex occurrence and development of LN. However, autophagy abnormality is only the manifestation of podocyte injury, and the relationship between autophagy abnormality and podocyte injury is not completely clear, and thus, requiring further research.

In the present study, HOXA11-OS was highly expressed in the kidney tissue and cells of lupus mice and in the blood of lupus patients. Overexpression of HOXA11-OS promoted the expression of Cyr61, thus enhancing serum immunoglobulin G (IgG)-induced podocyte autophagy in lupus patients and aggravating podocyte injury. Knocking down HOXA11-OS showed the opposite effect. In addition, as a ceRNA, HOXA11-OS regulated the expression of Cyr61 by sponging miR-124-3p. Based on these results, we hypothesize that the HOXA11-OS/miR-124-3p/Cyr61 regulatory network may be involved in the occurrence and development of LN by regulating podocyte autophagy. Therefore, HOXA11-OS was further investigated to verify this hypothesis and propose a new LN treatment strategy.

## Methods

### Reagents

The lentivirus and adeno-associated virus (AAV) were constructed at Shanghai Jikai Gene Chemistry Technology Co. Ltd. (Shanghai, China). Primers were synthesized by Shanghai Shenggong Biotechnology Company (Shanghai, China). The enhanced chemiluminescence kit, rabbit anti-glyceraldehyde 3-phosphate dehydrogenase (GAPDH) primary antibody (batch No.: 62u0922), rabbit anti-Cyr61 primary antibody (batch No.: 88 × 3333), mouse anti-tubulin primary antibody (batch No.: 6915213), goat anti-rabbit IgG secondary antibody (batch No.: 56j9958), and goat anti-mouse IgG secondary antibody (batch No.: 20000242) were purchased from Affinity Biosciences (Cincinnati, OH, USA). Rabbit anti-Beclin-1 primary antibody (batch No.: ab210498), rabbit anti-LC3B primary antibody (batch No.: ab192890), rabbit anti-nephrin primary antibody (batch No.: ab216341), and rabbit anti-podocin primary antibody (batch No.: ab181143) were obtained from Abcam (Cambridge, UK). Mouse anti-LC3B primary antibody (batch No.: K0420) was purchased from Santa Cruz Biotechnology Inc. (Dallas, TX, USA) and rabbit anti-β-actin primary antibody (batch No.: UD277186) from Invitrogen (Waltham, MA, USA). Goat anti-rabbit fluorescent antibody (batch No.: 211061011) and goat anti-mouse fluorescent antibody (batch No.: 216470915) were obtained from Beijing Zhongshan Jinqiao Biological Co. Ltd. (Beijing, China). The human IgG concentration detection kit (batch No.: TPFYJ7G4IY) was obtained from Elabscience Biotechnology Co. Ltd. (Houston, TX, USA). Enzyme-linked immunosorbent assay kits for mouse anti-double stranded DNA (dsDNA) antibody (batch No.: C05012542), mouse anti-nuclear antibody (ANA; batch No.: C05012540), and mouse anti-Smith (Sm) antibody (batch No.: C06012543) were purchased from CUSABIO Biotech Co. Ltd. (Houston, TX, USA). Cell Counting Kit-8 (CCK-8; batch No.: 20210828) was obtained from MCE (Suzhou, China) and the double luciferase detection kit (batch No.: E1910) from Beijing Promega Biotechnology Co. Ltd. (Beijing, China).

### Sample collection from patients

Serum samples were collected from 20 patients with diagnosed LN hospitalized in the Department of Nephrology and Immunology of Affiliated Hospital of Youjiang Medical College for Nationalities from July 2020 to August 2021. Sera from ten healthy people were collected as control samples. All participants signed informed consent forms. The collected serum samples were approved by the Ethics Committee of the Affiliated Hospital of Youjiang Medical College for Nationalities. The samples were used to detect the expression of HOXA11-OS, Cyr61, Beclin-1, and LC3B and to extract and purify IgG.

### Animal experiments

Four-month-old C57BL/6J female mice and MRL/lpr female mice were purchased from Jiangsu Changzhou Cavens Experimental Animal Co. Ltd. (License No.: SCXK (Su) 2016-0010; Changzhou, China). The mice were raised in the animal room of the Experimental Animal Center of Youjiang Medical College for Nationalities in a specific-pathogen-free environment, along with a 12 h/12 h light and dark cycle, ambient temperature of 22–24 ℃, and relative humidity of 60–70%. Animal experiments were approved by the Ethics Committee of Youjiang Medical College for Nationalities (Approval No.: 2020043001), and all procedures were carried out according to the guidelines of the National Institute of Health. All the experimental operations were carried out under anesthesia to alleviate the pain of experimental animals as much as possible.

Adeno-associated virus used in mouse kidney orthotopic injection was designed and constructed by Shanghai Jikai Gene Chemistry Technology Co., Ltd. According to the coding specificity of HOXA11-OS and miR-124-3p genes, the corresponding control sequences were obtained, and sh-HOXA11-OS and miR-124-3p inhibition sequences were designed according to the control sequences. The gene sequences used in this study are showed in Table 1. The experimental mice were randomly divided into eight groups, each with six mice: (1) Control group: normal mice injected with negative control AAV; (2) sh-HOXA11-OS group: normal mice injected with knockdown HOXA11-OS AAV; (3) miR-124-3p inhibition group: normal mice injected with miR-124-3p inhibition AAV; (4) sh-HOXA11-OS + miR-124-3p inhibition group: normal mice injected with knockdown HOXA11-OS AAV and miR-124-3p inhibition AAV; (5) Lupus group: lupus mice injected with negative control AAV; (6) Lupus + sh-HOXA11-OS group: lupus mice injected with knockdown HOXA11-OS AAV; (7) Lupus + miR-124-3p inhibition group: lupus mice injected with miR-124-3p inhibition AAV; and (8) Lupus + sh-HOXA11-OS + miR-124-3p inhibition group: lupus mice injected with knockdown HOXA11-OS AAV and miR-124-3p inhibition AAV. The concentration of AAV used was 5 × 10^11^ vg/mL, and the effects of AAV in all mice lasted for one month after in vivo injection.

**Table 1:** Gene sequences used in animal experiments

Gene	Sequences
sh-NC	CCATGATTCCTTCATATTTGC
sh-HOXA11-OS	ACCGGCCGGCAAGGCTATGACATTTTCAAGAGAAATGTCATAGCCTTGCCGGTTTT
miR-124-3p inhibition	ACCGGGCATTCACCGCGTGCCTTATTTT

### Cell culture and model establishment

The mouse podocyte cell line MPC5, donated by Professor Lin Xu of Youjiang Medical College for Nationalities, was cultured in RPMI-1640 medium containing 10% fetal bovine serum but not interferon-gamma in a cell incubator at 37 °C and 5% CO_2_. Lentivirus vectors used to transfect podocytes to achieve overexpression and knockdown of the target genes were designed and constructed by Shanghai Jikai Gene Chemistry Technology Co., Ltd. Stable cell lines were screened using puromycin (5 μg/mL, 48 h) in culture medium. According to the coding specificity of HOXA11-OS, miR-124-3p, and Cyr61 genes, the corresponding control sequences were obtained, and ex-HOXA11-OS, sh-HOXA11-OS, miR-124-3p mimic, miR-124-3p inhibition, and sh-Cyr61 sequences were designed according to the control sequences. The gene sequences used in this study are showed in Table 2.

**Table 2:** Gene sequences used in cells experiments

Gene	Sequences
ex-HOXA11-OS	GTCGGAGGAAGCGAGGTTTTCCGGGGTGCCGTAAGCCGTCTCGAAAAACTGGTCGAAAGCCTGTGGCAGAACGCCGTTCCTGCCCACCGTGCTATAGAAATTGGACGAGACGGCGGGGGTGGGGTGGTGGTAGACGTTGGCCGAGCTCTTGGCCAGCACGTCGCCAGGCACGCCGGCCGCGCTGGGCGCCTGCAGACAGTCTCTGTGCACGAGCTCCTCCGCGGAGTAGCAGTGGGCCAGATTGCCGCGGGGGTGCCATTTAGTGGCGGGCTCAATGGCGTACTCTCTGAAGGTCACTTCGCGCACGGGTTGGACCTGGGGCAGGTTGGAGGAGTAGGAGTATGTCATTGGGCGCGAAGACGGGGTCTGGGGCAAAAAAGAAGGGAGGCTGGAGAAATCTGGACCCGAGACGTAGTAAGTACAACTTGGCAAATACATGTTAGAGGAGCAGGGACCACGCTCATCAAAATCCATCATTGGGCTACCTTGGGCTCTCCGCAGTAGCCGAGCTTAACATGATTCTCCACTGCAGCTGCCTCTTTGAAGCGGATCCGTGAAGTAGAAATTTGGAGACCCACCTCAGGGGAAGCAACAGATCGTCACTCGGTGTTCTCACCGAAAGCACGTAATCGCCGGTGTAACTCATGTTGGCTGGGGGGCCTCCCCGCGCGCAGAAAGGCTGGGGTGCGCCCCCGGGCAGCTCTCCTTTGCTCAGCTACATGGTCCTGGTCCACGAGTGCTCTGAGGGCGGCAAGAGAGCGCAACTCCTGACGCCTCCCCCCACTCCCCGCCCCCCTCCCACAGGTTTGCTCTGGGAAGCCCCCCAGCCTCAGACCCTGGCTGGACCCCATTTGGGGCCAGGCTTCGCCGGCACGGATGTGCCGGCCTCGTGGCTTGTCCGATTTGCACGGTGACTTGATTACACGCTCTCATTCACGGTCACTTCCGAAGCGCTTTAGTGCCTTCCGTCCCCAAACCGCCAACAGGCAAAGCGGCTTCCCTCCGCGGGGTTCGGAGTGACTCCTCAGAGCCAGAGGCACTTCTGCTCACCGGTCCGCAAGCTGCCTGGTCTGCTGAAGCTGACGAATCGGGAAACCATGCAATTGAGGCGAACCTTGGGCTGTTTTAGAGGCGCTGAGGAGCCTTCTCCTGGGAGGCCCAAGGTTGATTTCAGCCCACCAGGATCTGGGGAAGACCCAACTAGGGATAAGAGCACACCAAAAGGCCAAGTCCGAGTTCCATTTCTAGAAGAGGCGGCTTCCGGCAAGGCTATGACATTGGCCCTGGACATTGGTTTCCCAGGAGCTGCTTTTTCTCAAGAACTCCACAGCACGGGGCTGTCTCCAGAAAACGCTCTTCAACGTTTATTTCTTTTAATCGTCGCCCGGAGCCCTAAGGCGGCTAATGCAAGAGGCCAAAAATGTTTGGAGGAAGAAAAACAAAGGCAGGAAGTGGCCGCGGCCTGACGGTGCGTGTGTGTCTGTAAAGAAGGGAGGGAGCCGGTTCAACCTCCCCTCGTTTTCCCGAACTTCAAGGTCTAGGCAGACCCCCTTAGGGCCTTGCCGAGGCTCGCCCCCACACCCCCAGCGGCGCAGCATTTGGAGGTGGCCAACGATTTAAGCCTCGGTCGGGCTGAAAGGAGATTTGATCGGCAGAACAAACCAACCCTTTTCGGAGGTTTCTTTTGATTTGGTCCTAAAGGGTATATGCTAGTGTCCACAGCGGCTGGGGTGGCTGCTGTTTTCCTCCCGCCGGGCTAAAAGTACCAAGAAGGGAGGGAGGGAGAGAGATTCAGGCACCTTGCGCTGGCTGCACTCTCCTTCTGAGATAGAATACCAG
ex-NC	GGGTCAATATGTAATTTTCAGTG
sh-HOXA11-OS#1	CCGGCCGGCAAGGCTATGACATTCTCGAGAATGTCATAGCCTTGCCGGTTTTT
sh-HOXA11-OS#2	CCGGGCTCTCCTTTGCTCAGCTACTCGAGTAGCTGAGCAAAGGAGAGCTTTTT
sh-HOXA11-OS#3	CCGGTTCCGACTGTACAGCCTATTCCTCGAGGAATAGGCTGTACAGTCGGAATTTTT
sh-NC	GGAAAGAATAGTAGACATAATAGC
sh-Cyr61#1	CCGGTTCCGACTGTACAGCCTATTCCTCGAGGAATAGGCTGTACAGTCGGAATTTTT
sh-Cyr61#2	CCGGTAACGAGAAACAATGAGTTAACTCGAGTTAACTCATTGTTTCTCGTTATTTTT
sh-Cyr61#3	CCGGCCTGTGAATATAACTCCAGAACTCGAGTTCTGGAGTTATATTCACAGGTTTTT
sh-NC	CCATGATTCCTTCATATTTGC
miR-124-3p mimic	CTTCCTTCTTTCTTTCCTTCCTTCCTTCTTCCTTCCTCAGGAGAAAGGCCTCTCTCTCCGTGTTCACAGCGGACCTTGATTTAAATGTCCATACAATTAAGGCACGCGGTGAATGCCAAGAATGGGGCTGTCTGAGCACCTTGGGTCCACGAGGGCCTGCCACGGAAGGATCGACCCAGCACAACGCCCTGGGCAGATCTCAGCGCTGCAGCTGCAGGCGCCCACAGTACTTTTTT
miR-124-3p inhibition	CCGGGGCATTCACCGCGTGCCTTATTTTTG
miR-NC	CCATGATTCCTTCATATTTGC

### Extraction and determination of serum IgG concentration

Serum IgG was separated and purified by protein affinity chromatography column elution, which included washing, loading column, equilibrium, loading sample, washing, elution, and neutralization. The concentration of IgG was determined according to the operation instructions of the kit.

### CCK-8 assay

Podocytes were induced with different concentrations of lupus serum IgG (250, 500, or 1000 μg/mL) for 3, 6, and 12 h after incubation for 24 h. The supernatant formed after induction was discarded and 10 μL CCK-8 solution was added to each well. After incubation for approximately 1 h, the optical density (OD) at 450 nm was measured using a microplate reader.

### Quantitative real time-polymerase chain reaction (qRT-PCR)

Total RNA was extracted in strict accordance with the instructions of the total RNA extraction kit. A reverse transcription kit was used to reverse transcribe RNA into complementary DNA. The SYBR Green qPCR kit was used for qRT-PCR detection using three replicates. The two-step amplification procedure was adopted, and the reaction conditions were as follows: pre-denaturation at 95 °C for 30 s (one cycle) and denaturation at 95 °C for 10 s and annealing and extension at 60 °C for 30 s (40 cycles). The relative expression of genes was calculated using the 2-ΔΔCt formula. U6 was used as the internal reference for miR-124-3p and β-actin as the internal reference for other genes. The sequences of the primers used in this experiment are shown in Table 3.

**Table 3:** qPCR primer sequences

Gene	Sequences (5’-3’)
H-HOXA11-OS-F	CTCTCCAAGCCCAGTTCA
H-HOXA11-OS-R	CAGCCCTCTTCCACCTC
H-Beclin-1-F	AACCAACGTCTTTAATGCAACCTTC
H-Beclin-1-R	AGCAGCATTAATCTCATTCCATTCC
H-LC3B-F	GATGTCCGACTTATTCGAGAGC
H-LC3B-R	TTGAGCTGTAAGCGCCTTCTA
H-Cyr61-F	ACTTCATGGTCCCAGTGCGC
H-Cyr61-R	AAATCCGGGTTTCTTTCACA
H-GAPDH-F	CAGGAGGCATTGCTGATGAT
H-GAPDH-R	GAAGGCTGGGGCTCATTT
m-HOXA11-OS-F	GCACGTAATCGCCGGTGTAA
m-HOXA11-OS-R	CTGAGCAAAGGAGAGCTGCC
m-Beclin-1-F	ATTGAAGACACTGGAGGCA
m-Beclin-1-R	CAGGCAAGACCCCACTT
m-LC3B-F	CACCACCCACAGATGAGA
m-LC3B-R	AGACGAGTTTCCAAGCTGA
m-nephrin-F	TGTGCTGGTGATGACCGTTC
m-nephrin-R	AGAGTGATGCTTCGCTCCTG
m-podocin-F	GATGGCGGCTGAGATTCTGT
m-podocin-R	GTGGTTCAACTGGTTTGGAGG
m-Cyr61-F	GGATCTGTGAAGTGCGTCCT
m-Cyr61-R	CTGCATTTCTTGCCCTTTTT
m-β-actin-F	CCTCACTGTCCACCTTCC
m-β-actin-R	GGGTGTAAAACGCAGCTC
m-miR-124-3p-F	GCTAAGGCACGCGGTG
m-miR-124-3p-R	GTGCAGGGTCCGAGGT
m-miR-124-3p-RT	GTCGTATCCAGTGCAGGGTCCGAGG
	TATTCGCACTGGATACGACGGCATT
m-U6-F	GCTTCGGCAGCACATATACTAAAAT
m-U6-R	CGCTTCACGAATTTGCGTGTCAT

### Fluorescence in situ hybridization (FISH)

Cells fixed on slides were permeated with 0.5% Triton X-100 [0.1 M in phosphate-buffered saline (PBS)] for 10 min and then incubated in a pre-hybridization solution (without probe) on a climbing piece (floating method). Pre-hybridization was performed at 37 °C in a sealed wet box for 30 min–2 h. After rinsing, first at 42 °C and then at room temperature, 4’,6-diamidino-2-phenylindole (DAPI) was added dropwise and cells were incubated for 5 min. Excess DAPI was removed by washing four times with PBS plus Tween 20 for 5 min each time. A sealing sheet liquid containing an anti-fluorescence quencher was applied before fluorescence microscopy observation.

### Double luciferase gene reporting assay

The binding sites of miR-124-3p to HOXA11-OS and Cyr61 were analyzed by bioinformatics analysis. The activity of luciferase was determined using the double luciferase detection kit. The fragments of HOXA11-OS and Cyr61 bound to miR-124-3p were amplified by qRT-PCR, and then inserted into the pMIR-REPORT vector to construct wild-type plasmids of HOXA11-OS and Cyr61. The binding sites were then mutated by gene mutation technology to construct mutant plasmids of HOXA11-OS and Cyr61. 293 T cells were transfected with HOXA11-OS and Cyr61 wild type or mutant plasmids and miR-124-3p mimics, either alone or in combination. After 24 h, luciferase activity was analyzed using the double luciferase reporter detection system.

### Renal function measurement

Serum creatinine (Scr), urea nitrogen (BUN), and 24 h urine protein levels were analyzed to evaluate renal function using the Scr, BUN, and 24 h urine protein detection kits, respectively, following the manufacturers’ instructions.

### Enzyme-linked immunosorbent assay

The levels of anti-dsDNA, ANA, and anti-Sm antibodies in mouse serum were detected using the respective enzyme-linked immunosorbent assay kits. The absorbance was measured at 450 nm using a microplate reader.

### Hematoxylin–eosin and periodate-Schiff staining

The kidney tissues of mice were fixed with 4% paraformaldehyde fixation solution, embedded with paraffin wax, and then sectioned continuously (approximately 4 μm in thickness). Dewaxing and hydration were performed before hematoxylin–eosin and periodate-Schiff staining for optical microscopy analysis. Renal pathology examinations were performed by a pathologist blinded to relevant experimental criteria.

### Western blotting

Mouse kidney tissues and podocytes were lysed in RIPA lysis solution and then centrifuged at 4 °C and 12,000 rpm for 5 min to collect the supernatant. Protein concentration was detected using the bicinchoninic acid assay. After sodium dodecyl-sulfate polyacrylamide gel electrophoresis, the denatured protein was transferred to a polyvinylidene difluoride membrane, which was sealed in Quick Block™ Western sealing solution at room temperature for 10 min. Cyr61 (1:1,500), Beclin-1 (1:2,000), LC3B (1:2,000), nephrin (1:1,500), podocin (1:2,000), GAPDH (1:6,000), and β-actin (1:6.000) were then added and incubated overnight in a shaking table at 4 °C. The membrane was reacted with horseradish peroxidase secondary antibody (1:20,000), developed using enhanced chemiluminescence reagent, and analyzed using ImageJ 1.8.0 (National Institutes of Health, Bethesda, MD, USA) to calculate the expression of each protein.

### Immunofluorescence (IF) assay

Podocytes (1 × 10^4^ cells/well) were inoculated into confocal culture dishes for 24 h, and 4% paraformaldehyde cell fixation solution was added for 30 min. The cells were permeated with 0.1% TritonX-100-PBS for 20 min and then sealed with 10% bovine serum albumin for 30 min. The primary antibodies of Cyr61 (1:200), LC3B (1:800), nephrin (1:200), and tubulin (1:200) were added and incubated overnight at 4 °C. After further incubation with fluorescent secondary antibodies (1:200) in the dark for 1 h, 1–2 drops of DAPI were added for nuclear staining for 10 min, followed by anti-fluorescence quencher addition.

Mouse kidney tissue sections were dewaxed using dimethylbenzene, dehydrated in a graded ethanol series, and boiled in ethylenediaminetetraacetic acid buffer (pH 8.0) for 20 min for antigen repair. For sealing, 10% bovine serum albumin was added for 1 h. Cyr61 (1:200), LC3B (1:800), and nephrin (1:200) were then added and tissue sections were further incubated overnight. After incubation with fluorescent secondary antibody (1:200) for 1 h, 1–2 drops of DAPI were added for nuclear staining for 10 min, and the anti-fluorescent quenching tablets were sealed. The laser confocal microscope was used to analyze the samples.

### Statistical analyses

Data were analyzed in SPSS 23.0 (IBM, Armonk, NY, USA) and GraphPad Prism 8.0 (GraphPad Software, San Diego, CA, USA). All data are expressed as means ± standard deviation. The independent samples *t*-test was used for comparing two groups. One-way analysis of variance was used for comparing multiple groups, and the least significant difference test was used for comparing groups when variance was homogeneous. The Tamhane's T^2^ test was used when the variance was not uniform. *P* < 0.05 indicates statistical significance.

## Results

### Increased expression of HOXA11-OS, Cyr61, Beclin-1, and LC3B in the kidney tissue of lupus mice and serum of lupus patients

**Fig. 1:**
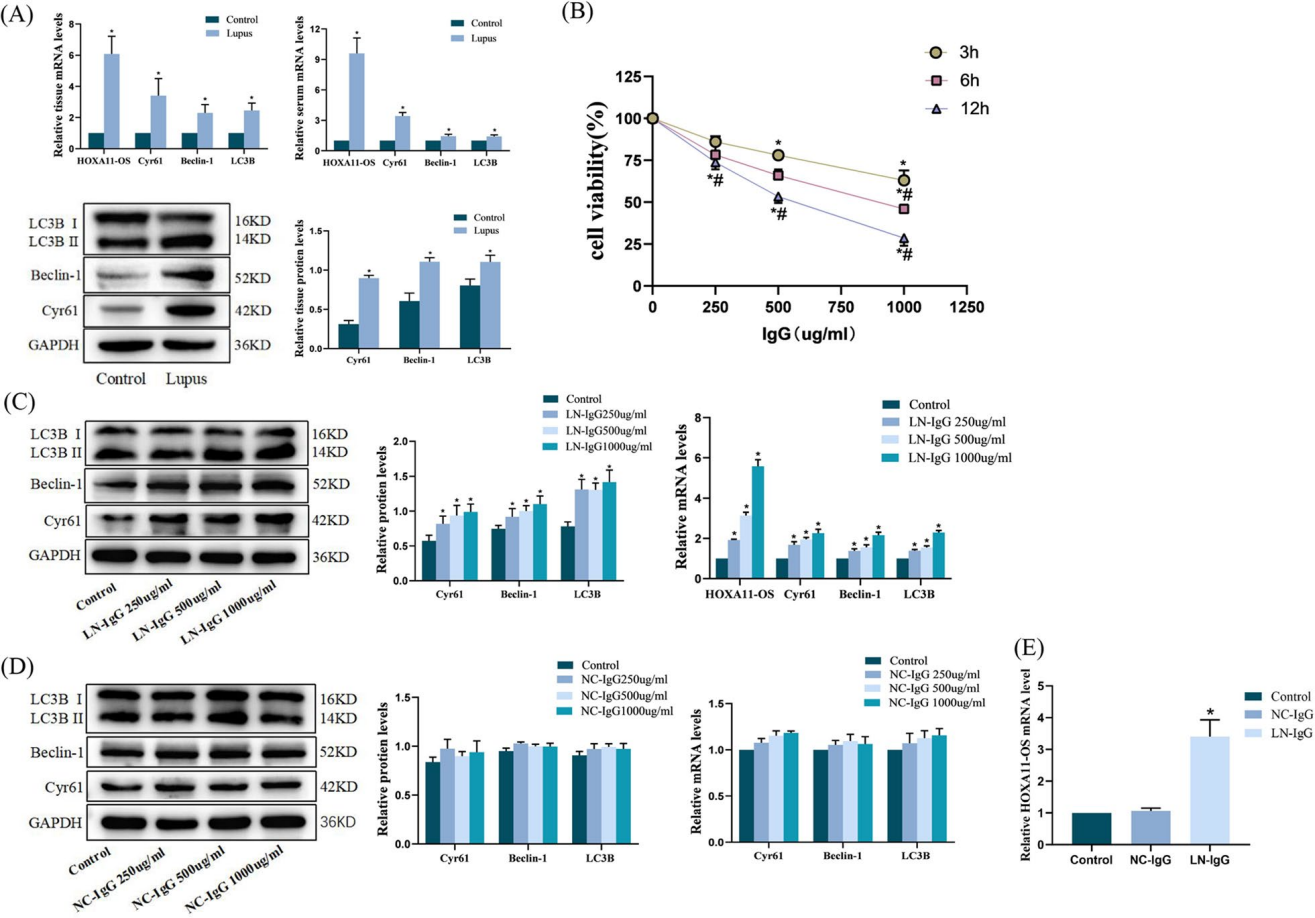
HYPERLINK "sps:id::fig1||locator::gr1||MediaObject::0" HOXA11-OS, Cyr61, Beclin-1, and LC3B are highly expressed in kidney tissues, serum, and cell lines of LN. **A** Expression of HOXA11-OS, Cyr61, Beclin-1, and LC3B in the kidney tissue of lupus mice and serum of lupus patients as detected by qRT-PCR and western blotting. ^*^P < 0.05 vs. control group. Data are presented as means ± standard deviation. **B** Podocytes induced by different concentrations of serum IgG (250, 500, or 1000 μg/mL) from lupus patients for 3, 6, and 12 h. Podocyte activity was detected by CCK-8. ^*^P < 0.05 vs. 0 μg/mL; ^#^*P* < 0.05 vs. 3 h. **C** Expression of HOXA11-OS, Cyr61, Beclin-1, and LC3B as detected by qRT-PCR and western blotting after podocytes were induced by different concentrations of IgG from lupus patients sera for 6 h. ^*^P < 0.05 vs. control group. **D** Expression of Cyr61, Beclin-1, and LC3B as detected by qRT-PCR and western blotting after podocytes were induced by different concentrations of IgG from normal human serum for 6 h. **E** Expression of HOXA11-OS mRNA in control, NC-IgG (1000 μg/mL, 6 h) and LN-IgG (1000 μg/mL, 6 h) groups was detected by qRT-PCR. ^*^P < 0.01 vs. control group

The expression of HOXA11-OS, Cyr61, and the autophagy factors Beclin-1 and LC3B in the kidney tissue of lupus mice and serum of lupus patients was detected by qRT-PCR and western blotting. Compared with the control group, the expression of HOXA11-OS, Cyr61, Beclin-1, and LC3B was significantly enhanced (*P* < 0.05) (Fig. 1A).

### Serum IgG from lupus patients induces the expression of HOXA11-OS, Cyr61, Beclin-1, and LC3B in podocytes in a concentration-dependent manner

To establish a cell model, podocytes were induced with different concentrations of serum IgG from lupus patients (LN-IgG 250, 500, or 1000 μg/mL) for 3, 6, and 12 h. The results showed that cell viability decreased gradually with increased induction time and dose, and the half inhibitory concentration appeared at induction using IgG 1000 μg/mL for 6 h (Fig. 1B). The results showed that LN-IgG could inhibit the activity of podocytes, and podocytes induced at 1000 μg/mL LN-IgG for 6 h were used in the follow-up experiments.

According to the effects of LN-IgG on podocyte viability under the different conditions, we induced podocytes with different concentrations of LN-IgG for 6 h, and then collected them for qRT-PCR and western blot analyses. The expression of HOXA11-OS, Cyr61, Beclin-1, and LC3B were significantly increased, and the increase was most obvious when podocytes were induced by IgG at 1000 μg/mL for 6 h (*P* < 0.05) (Fig. 1C).

Subsequently, the effects of podocyte induction with LN-IgG or serum IgG for 6 h on the expression of Cyr61, Beclin-1, and LC3B were compared using the same method and different normal serum IgG concentrations (NC-IgG 250, 500, or 1000 μg/mL). There were no significant differences in the expression of Cyr61, Beclin-1, and LC3B (*P* > 0.05) between LN-IgG- and NC-IgG-induced groups (Fig. 1D). According to the above experimental basis, we detected the expression of HOXA11-OS in cells of Control, NC-IgG (1000 μg/mL, 6 h) and LN-IgG (1000 μg/mL, 6 h) groups by qRT-PCR. The results showed that, compared with the Control group, there was no significant difference in HOXA11-OS mRNA expression in NC-IgG group (*P* > 0.05), while the mRNA expression level of HOXA11-OS in LN-IgG group was significantly increased (*P* < 0.01) (Fig. 1E). These results suggest that serum IgG of lupus patients may regulate the expression of HOXA11-OS, Cyr61 and autophagy factors in podocytes.

### HOXA11-OS regulates the expression of Cyr61 and autophagy and podocyte markers in podocytes

**Fig. 2:**
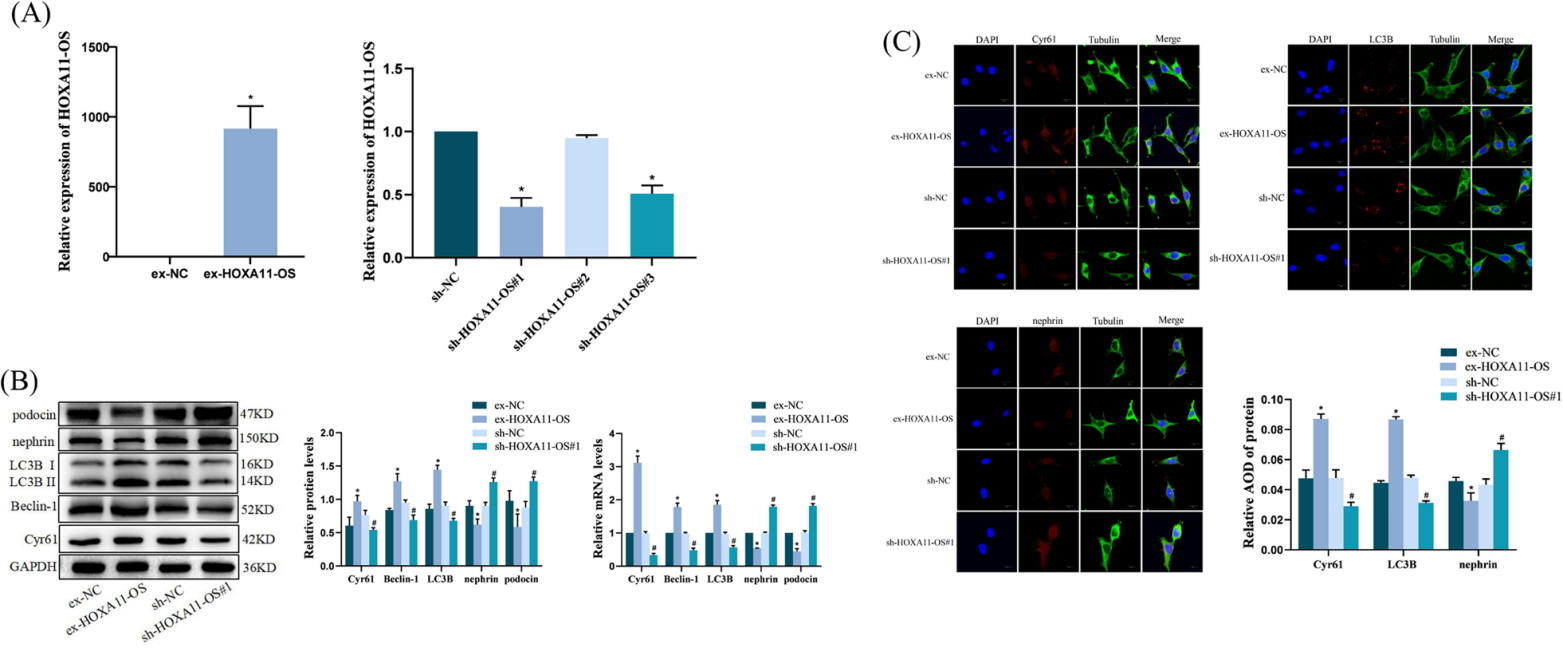
Expression of Cyr61, autophagy factors, and podocyte markers in podocytes regulated by HOXA11-OS. **A** HOXA11-OS mRNA expression was detected by qRT-PCR after HOXA11-OS overexpression and knockdown lentiviruses were successfully transfected into podocytes. ^*^P < 0.001 vs. ex-NC; ^*^P < 0.001 vs. sh-NC. **B** After HOXA11-OS overexpression and knockdown lentiviruses were successfully transfected into podocytes, the expression of Cyr61, Beclin-1, LC3B, nephrin, and podocin was detected by qRT-PCR and western blotting. ^*^P < 0.01 vs. ex-NC; ^#^*P* < 0.01 vs. sh-NC. C After HOXA11-OS overexpression and knockdown lentiviruses were successfully transfected into podocytes, the localization and expression of Cyr61, LC3B, and nephrin proteins in each group were detected by IF. Relative average optical density; ^*^P < 0.01 vs. ex-NC; ^#^*P* < 0.01 vs. sh-NC. Scale bars represent 15 µm

We successfully constructed HOXA11-OS overexpression and knockdown stable cell lines after lentivirus transfection of podocytes and verified HOXA11-OS expression by qRT-PCR. The expression of HOXA11-OS was significantly increased in the ex-HOXA11-OS group (*P* < 0.001), while that of HOXA11-OS#1 and #3 was significantly decreased in the sh-HOXA11-OS group. HOXA11-OS#1 decreased most significantly, and the knockdown effect was the most pronounced (*P* < 0.001). Hence, HOXA11-OS#1 was selected for further experiments (Fig. 2A).

qRT-PCR and western blot analyses were also used to detect the expression of Cyr61, Beclin-1, LC3B, and the podocyte markers nephrin and podocin. Compared with ex-NC, the expression of Cyr61, Beclin-1, and LC3B in the ex-HOXA11-OS group was significantly increased, while that of nephrin and podocin was significantly decreased (*P* < 0.01). Compared with sh-NC, the expression of Cyr61, Beclin-1, and LC3B in the sh-HOXA11-OS#1 group was significantly decreased, while that of nephrin and podocin was significantly increased (*P* < 0.01) (Fig. 2B).

In addition, the localization and expression of Cyr61, LC3B, and nephrin proteins were observed by IF. Cyr61, as a secretory protein, was expressed in the nucleus and cytoplasm, nephrin was expressed in the cell membrane, and LC3B was expressed in the cytoplasm. The expressions of Cyr61, LC3B, and nephrin obtained by IF were consistent with those obtained by western blotting (Fig. 2C). These results suggest that HOXA11-OS may affect podocyte function by regulating Cyr61 expression and autophagy.

### miR-124-3p directly targets the 3' untranslated region of HOXA11-OS and Cyr61 in podocytes

**Fig. 3:**
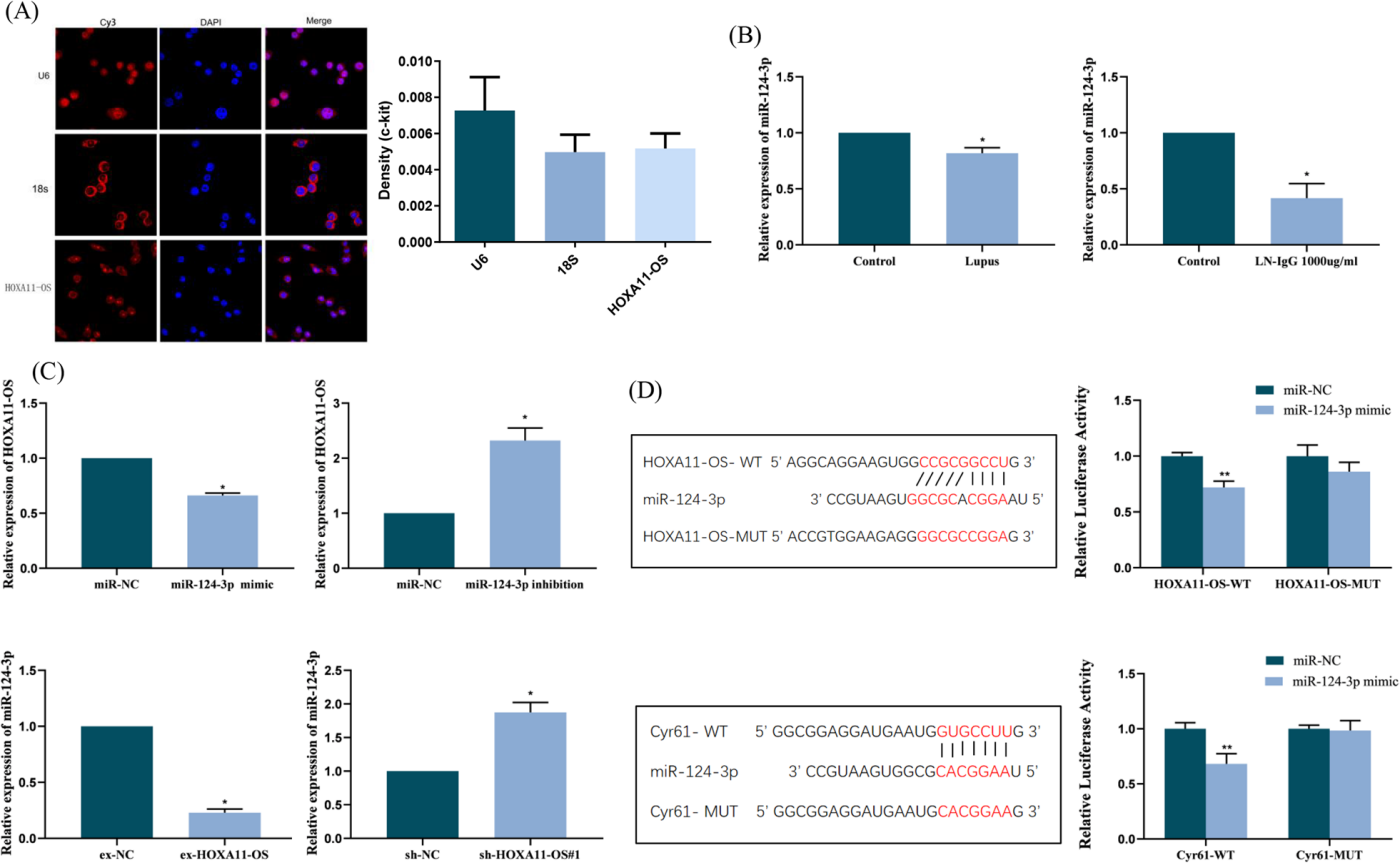
miR-124-3p directly binds to the 3’ untranslated regions of HOXA11-OS and Cyr61 in podocytes. **A** The subcellular localization of HOXA11-OS was detected by FISH assay. The expression of 18S, U6, and HOXA11-OS were similar, and there was no statistical difference in optical density. **B** The expression of miR-124-3p in the kidney tissue of lupus mice and podocyte injury model was detected by qRT-PCR. ^*^P < 0.01 vs. control group. **C** The expression of HOXA11-OS in miR-124-3p mimic or inhibition cell lines and the expression of miR-124-3p in HOXA11-OS overexpression or knockdown cell lines were detected by qRT-PCR. ^*^P < 0.001 vs. miR-NC/ex-NC/sh-NC. **D** The binding sites of miR-124-3p on HOXA11-OS and Cyr61 in HOXA11-OS-wild-type (WT) and Cyr61-WT, and the mutation sites in HOXA11-OS-mutant (MUT) and Cyr61-MUT are indicated in red. Bi-luciferase reporter genes were detected 48 h after transfection of HOXA11-OS-WT, Cyr61-WT, HOXA11-OS-MUT, or Cyr61-MUT and miR-124-3p mimic or miR-NC. ^**^P < 0.01 vs. HOXA11-OS-WT + miR-NC/Cyr61-WT + miR-NC

Firstly, the subcellular localization of HOXA11-OS was detected by FISH. The expressions of 18S, U6, and HOXA11-OS were similar, and there was no significant difference in optical density (*P* > 0.05). HOXA11-OS was distributed in both the nucleus and cytoplasm but mainly in the cytoplasm (Fig. 3A). In addition, qRT-PCR results showed that miR-124-3p expression was low in both the kidney tissue of lupus mice and podocyte injury model when compared with the control group (*P* < 0.05) (Fig. 3B). In the podocyte injury model, overexpression of HOXA11-OS significantly decreased the expression of miR-124-3p, while HOXA11-OS knockdown significantly increased the expression of miR-124-3p (*P* < 0.001). Furthermore, the overexpression of miR-124-3p significantly decreased the expression of HOXA11-OS, while miR-124-3p knockdown significantly enhanced the expression of HOXA11-OS (*P* < 0.001) (Fig. 3C). These results indicate that the expression of HOXA11-OS is negatively correlated with that of miR-124-3p and that HOXA11-OS may regulate miR-124-3p as a ceRNA.

Data in the Bibiserv database (https://bibiserv.cebitec.uni-bielefeld.de/rnahy Brid? ID = rnahybrid_view_submission) and Targetscan database (http://www.target-scan.org) predicted miR-124-3p targeted binding sites in both HOXA11-OS and Cyr61. The double luciferase reporter gene assay showed that the miR-124-3p mimic significantly reduced luciferase activity in cells transfected with the HOXA11-OS or Cyr61 wild type reporter gene but had almost no inhibitory effect on cells transfected with HOXA11-OS or Cyr61 mutant reporter gene (Fig. 3D). In conclusion, miR-124-3p can bind directly to the predicted binding sites of HOXA11-OS and Cyr61.

### HOXA11-OS mediates Cyr61 regulation of autophagy factors and cell damage by targeting miR-124-3p

**Fig. 4:**
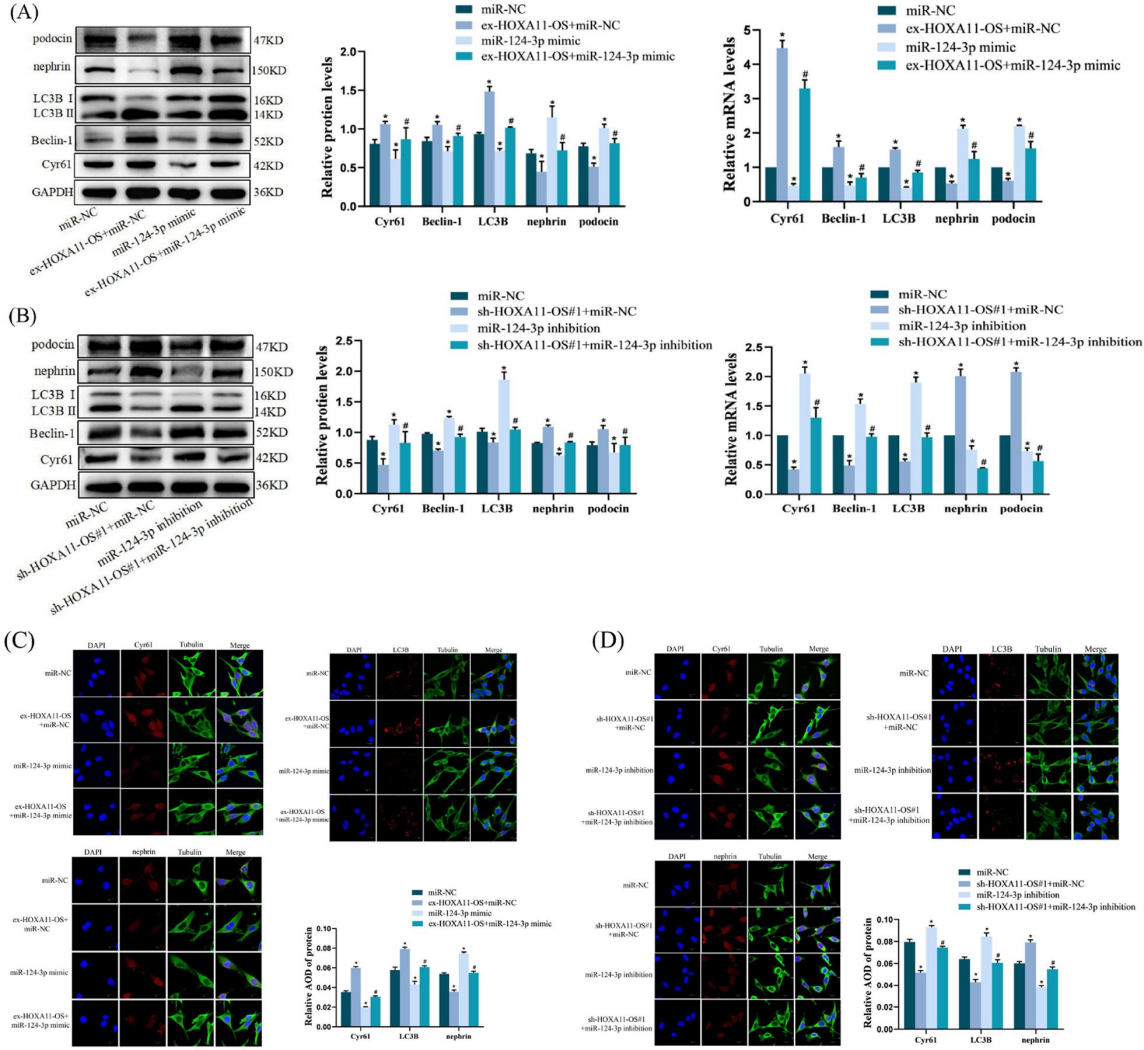
HOXA11-OS regulates autophagy factors and cell damage by targeting miR-124-3p mediating Cyr61. **A** The expression of Cyr61, Beclin-1, LC3B, nephrin, and podocin was detected by qRT-PCR and western blotting after transfection of miR-124-3p mimic into the ex-HOXA11-OS cell line. **B** The expression of Cyr61, Beclin-1, LC3B, nephrin, and podocin was detected by qRT-PCR and western blotting after transfection of miR-124-3p inhibition into the sh-HOXA11-OS#1 cell line. **C**, **D** The localization and expression of Cyr61, LC3B, and nephrin were detected by IF. ^*^P < 0.05 vs. miR-NC; ^#^*P* < 0.05 vs. ex-HOXA11-OS + miR-NC/sh-HOXA11-OS#1 + miR-NC. Data are presented as means ± standard deviation. Scale bars represent 15 µm

The experiments described above showed that the expression of miR-124-3p was opposite to that of HOXA11-OS and Cyr61, both containing direct targeting binding sites of miR-124-3p. This suggested that HOXA11-OS could mediate Cyr61 to regulate autophagy factor expression in podocytes by sponging miR-124-3p, thereby affecting podocyte function. To validate this hypothesis, a miR-124-3p-overexpressing lentivirus was transfected into the HOXA11-OS stable cell line. Cyr61 expression, levels of autophagy factors, and podocyte injury were detected by qRT-PCR and western blotting. The overexpression of Cyr61 and abnormally elevated levels of the autophagy factors Beclin-1 and LC3B induced by HOXA11-OS overexpression were downregulated, the podocyte marker proteins nephrin and podocin were upregulated, and podocyte injury was alleviated by miR-124-3p overexpression (*P* < 0.05) (Fig. 4A). After transfection of miR-124-3p knockdown lentivirus into the constructed knockdown HOXA11-OS stable cell line, the low expression of Cyr61 and the decreased levels of Beclin-1 and LC3B due to HOXA11-OS knockdown were reversed to some extent, while nephrin and podocin were downregulated and podocyte injury was aggravated (*P* < 0.05) (Fig. 4B). In addition, we observed the localization and expression of Cyr61, LC3B, and nephrin proteins by IF. The localization of Cyr61, LC3B, and nephrin proteins was the same as described above, and the protein expression trend was consistent with that detected by western blotting (Fig. 4C, D).

### Downregulation of Cyr61 can reverse the abnormal autophagy and podocyte injury induced by HOXA11-OS

**Fig. 5:**
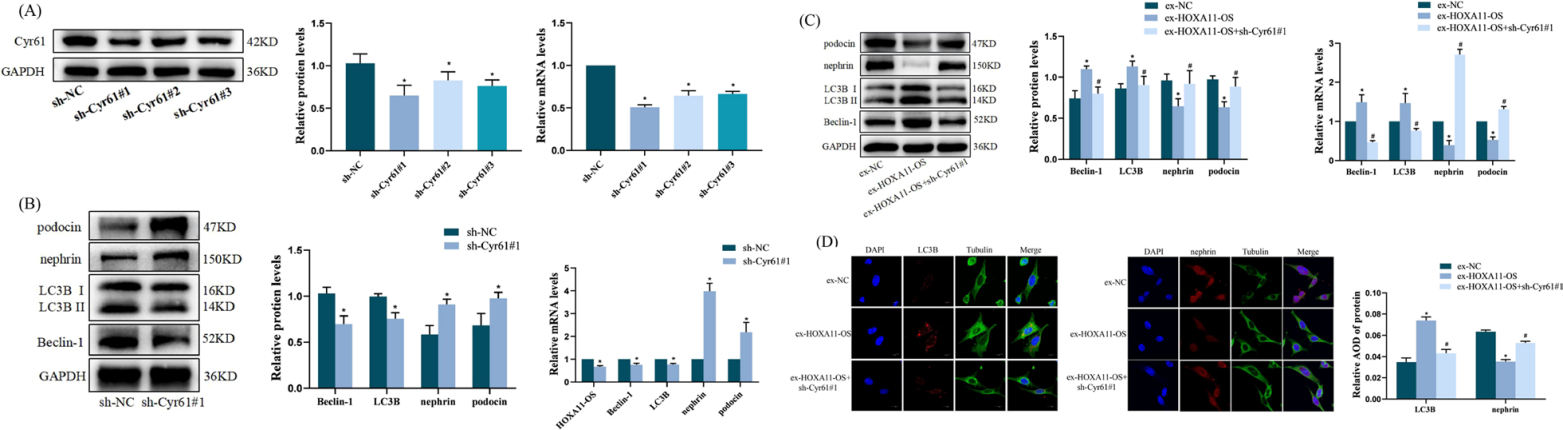
The downregulation of Cyr61 gene can reverse the abnormal autophagy and podocyte injury induced by HOXA11-OS. **A** The Cyr61 gene was downregulated by knockdown lentivirus transfected into podocytes, and its expression was detected by qRT-PCR and western blotting. **B** The expression of HOXA11-OS, Beclin-1, LC3B, nephrin, and podocin was detected by qRT-PCR and western blotting after Cyr61 downregulation. ^*^P < 0.05 vs. sh-NC. **C** The expression of Beclin-1, LC3B, nephrin, and podocin was detected by qRT-PCR and western blotting after sh-Cyr61#1 was transfected into the ex-HOXA11-OS cell line. **D** The localization and expression of LC3B and nephrin were detected by IF. ^*^P < 0.05 vs. ex-NC; ^#^*P* < 0.05 vs. ex-HOXA11-OS. Data are presented as means ± standard deviation. Scale bars represent 15 µm

In the present study, the expression of Cyr61 was downregulated by knocking down the lentivirus-transfected podocytes (Fig. 5A). The qRT-PCR and western blotting results revealed that the expression levels of HOXA11-OS, Cyr61, Beclin-1, and LC3B were significantly decreased, while nephrin and podocin were significantly increased in stable podocyte lines with downregulated Cyr61 (Fig. 5B). These results suggest that Cyr61 downregulation may reduce podocyte injury by downregulating the high expression of HOXA11-OS and reducing the abnormally elevated podocyte autophagy. Subsequently, the Cyr61 knockdown lentivirus was transfected into the constructed HOXA11-OS overexpressed stable cell line, and the expression level of the target gene was detected by qRT-PCR, western blot, and IF analyses. The results showed that the abnormal increase of autophagy caused by HOXA11-OS overexpression could be reversed to some extent, and the expression levels of Beclin-1 and LC3B decreased significantly, while that of nephrin and podocin increased significantly (*P* < 0.05); podocyte injury was alleviated (Fig. 5C, D). The recovery validation test showed that Cyr61 downregulation could alleviate the abnormally elevated autophagy of podocytes induced by the overexpression of HOXA11-OS and reduce podocytes damage.

### miR-124-3p inhibition leads to the deterioration of lupus mice with relieved injury after HOXA11-OS knockdown

**Fig. 6:**
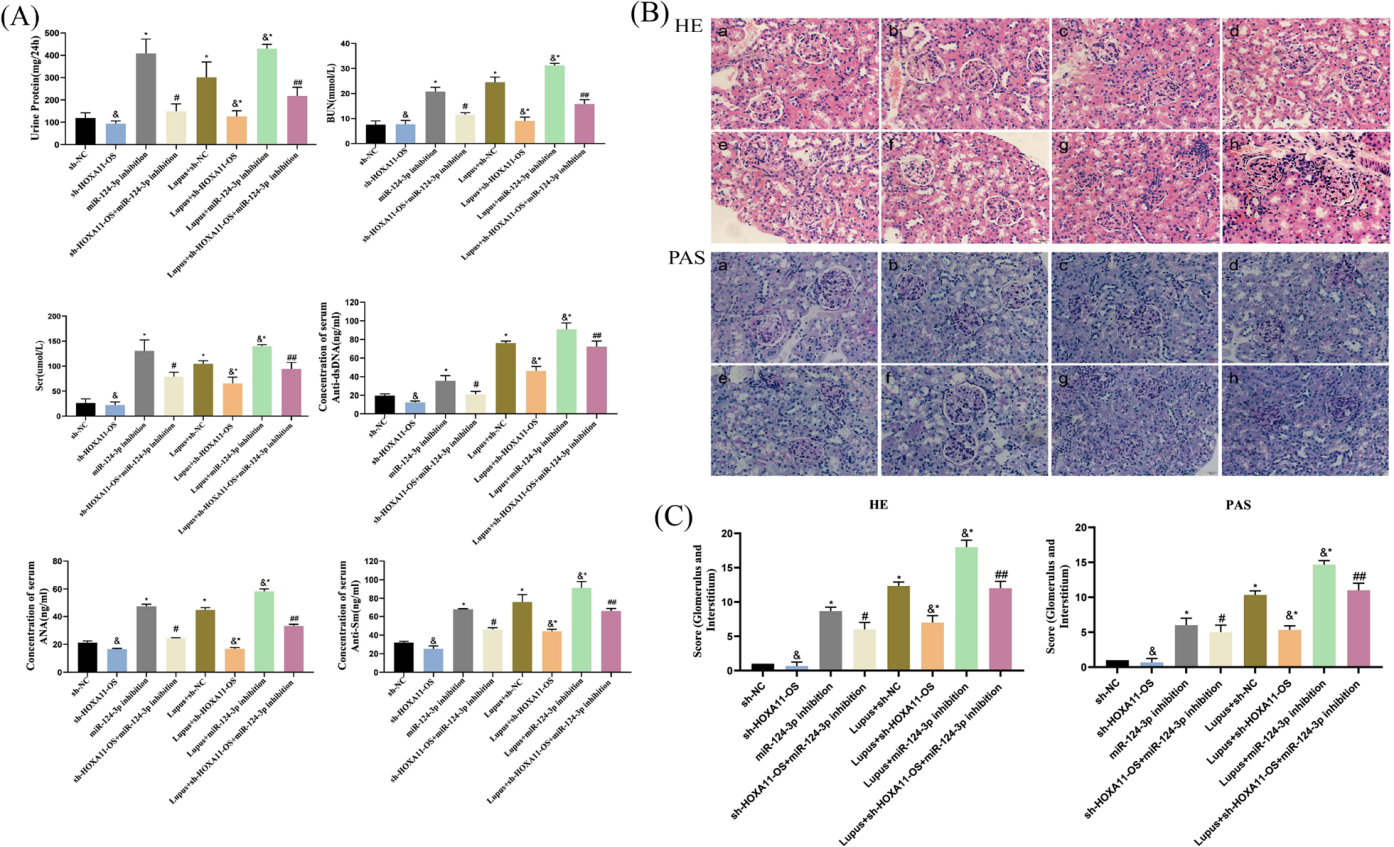
miR-124-3p inhibition leads to the deterioration of lupus mice but podocyte injury was alleviated after HOXA11-OS knockdown. Levels of 24 h urinary protein, BUN, Scr, and autoantibody in mice in each group. ^&^*P* > 0.05, ^*^P < 0.05 vs. sh-NC; ^#^*P* < 0.05 vs. sh-HOXA11-OS; ^&*^P < 0.05 vs. Lupus + sh-NC; ^##^*P* < 0.05 vs. Lupus + sh-HOXA11-OS. B,C hematoxylin–eosin and periodate-Schiff staining and histological score of kidney tissues of different groups: **a** sh-NC; **b** sh-HOXA11-OS; **c** miR-124-3p inhibition; **d** sh-HOXA11-OS + miR-124-3p inhibition; **e** Lupus + sh-NC; **f** Lupus + sh-HOXA11-OS; **g** Lupus + miR-124-3p inhibition; and **h** Lupus + sh-HOXA11-OS + miR-124-3p inhibition. Scale bars represent 20 µm. ^&^*P* > 0.05, ^*^P < 0.05 vs. sh-NC; ^#^*P* < 0.05 vs. sh-HOXA11-OS; ^&*^P < 0.05 vs. Lupus + sh-NC; ^##^*P* < 0.05 vs. Lupus + sh-HOXA11-OS

To further verify the molecular mechanism of HOXA11-OS in the occurrence and development of LN in vivo, sh-HOXA11-OS and miR-124-3p inhibition AAVs were injected into mouse kidneys, and changes in the renal function and autoantibody levels were detected. The levels of 24 h urinary protein, BUN, Scr, as well as anti-dsDNA, ANA, and anti-Sm antibodies in the Lupus + sh-HOXA11-OS group were significantly lower than those in the Lupus group (*P* < 0.05), while those in the Lupus + miR-124-3p inhibition group were significantly higher (*P* < 0.05). After treatment with both sh-HOXA11-OS and miR-124-3p inhibition AAV, the levels of 24 h urinary protein, BUN, Scr, as well as the levels of anti-dsDNA, ANA, and anti-Sm antibodies in mice were significantly higher than those in the Lupus + sh-HOXA11-OS group (*P* < 0.05) (Fig. 6A).

To further evaluate renal injury, Austin histological scores were used to assess global glomerular abnormalities and interstitial inflammation by examining HE- and PAS-stained renal tissue sections. The results showed contraction and deformation of glomeruli at different degrees, glomerular capillary stenosis and occlusion, mesangial cell and endothelial cell proliferation, and inflammatorcell infiltration in the renal interstitium in the Lupus group compared with the control group. After injection of sh-HOXA11-OS AAV, pathological lesions in the kidney tissue of mice in the Lupus + sh-HOXA11-OS group were significantly improved compared with those of mice in the Lupus group; the morphology of glomeruli was relatively normal, there was no obvious stenosis and occlusion of glomerular capillaries, the proliferation of mesangial cells and endothelial cells was reduced, and the number of infiltrated inflammatory cells was also decreased significantly. After injection of miR-124-3p inhibition AAV, the pathological lesions in the renal tissue of mice in the Lupus + sh-HOXA11-OS + miR-124-3p inhibition group were further aggravated compared to those of mice in the Lupus + sh-HOXA11-OS group. These results indicate that HOXA11-OS is involved in renal pathological processes and knocking down HOXA11-OS can alleviate renal injury in lupus mice; however, knocking down miR-124-3p may aggravate renal function injury in lupus mice by reversing the effect of HOXA11-OS knockdown (Fig. 6B, C).

### Effect of HOXA11-OS on the expression of autophagy factors and podocyte markers in lupus mouse renal tissues by targeting miR-124-3p regulating Cyr61

**Fig. 7:**
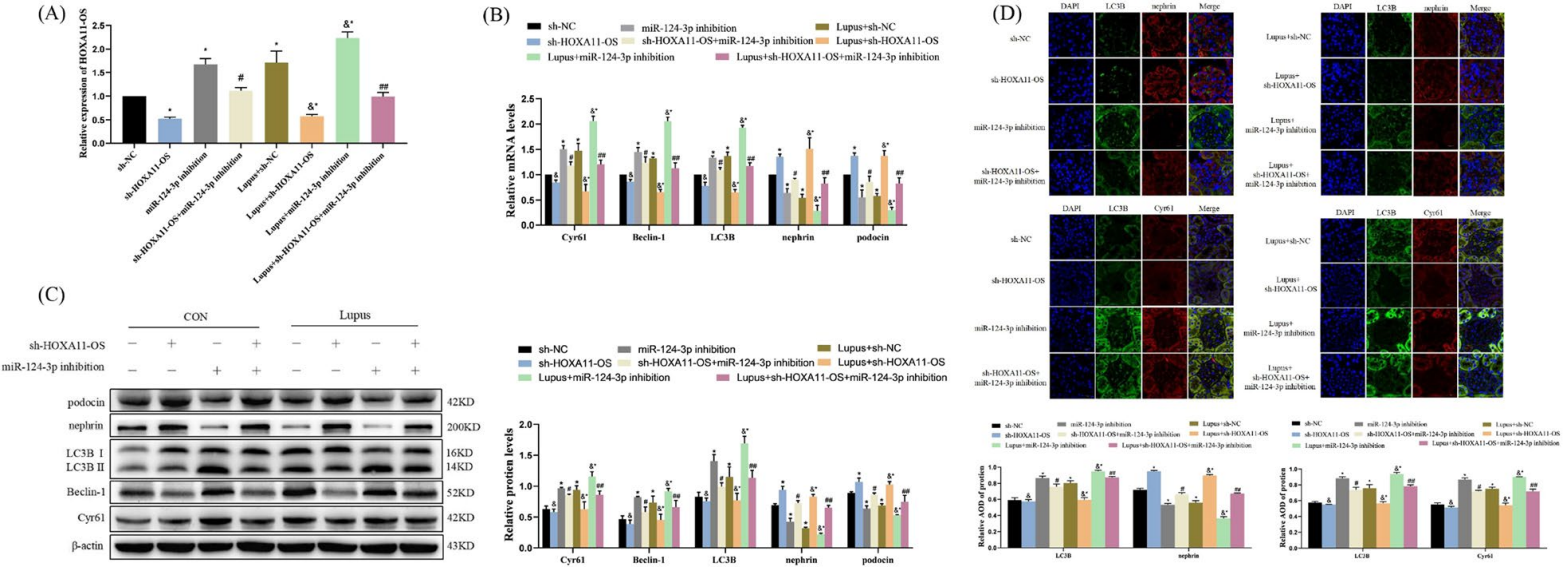
Effect of HOXA11-OS on the expression of autophagy factors and podocyte markers in kidney tissues by targeting miR-124-3p regulating Cyr61. **A** The expression of HOXA11-OS mRNA was detected by qRT-PCR after AAV was injected into mouse kidney tissues. **B** The expression of Cyr61, Beclin-1, LC3B, nephrin, and podocin in kidney tissues of mice was detected by qRT-PCR and western blotting. **C** The expression of Cyr61, LC3B, and nephrin in kidney tissues of mice was detected by IF. ^&^*P* > 0.05, ^*^P < 0.05 vs. sh-NC; ^#^*P* < 0.05 vs. sh-HOXA11-OS; ^&*^P < 0.05 vs. Lupus + sh-NC; ^##^*P* < 0.05 vs. Lupus + sh-HOXA11-OS. Data are presented as means ± standard deviation. Scale bars represent 15 µm

The knockdown level of HOXA11-OS in mouse kidney tissues was detected by qRT-PCR after in situ injection of AAV (Fig. 7A). Our results confirmed that HOXA11-OS is highly expressed in LN, and the expression of HOXA11-OS can be downregulated by injecting AAV in vivo. The qRT-PCR and western blot analyses revealed that the expression of Cyr61, Beclin-1, and LC3B in lupus mice was significantly decreased (*P* < 0.01), while that of nephrin and podocin was significantly increased (*P* < 0.05) after HOXA11-OS knockdown compared with the control group, suggesting that renal inflammatory lesions were alleviated. The expression of Cyr61, Beclin-1, and LC3B was significantly increased (*P* < 0.01), while that of nephrin and podocin was significantly decreased (*P* < 0.05) after miR-124-3p expression was downregulated, suggesting that renal inflammatory lesions were aggravated. Compared with the Lupus + sh-HOXA11-OS group, the expression of Cyr61, Beclin-1, and LC3B in the kidney tissue of mice in the control group was significantly increased (*P* < 0.05), while nephrin and podocin expression was significantly decreased (*P* < 0.01) after knockdown HOXA11-OS and knockdown miR-124-3p AAVs were injected simultaneously, reversing the effect of HOXA11-OS knockdown and aggravating kidney damage (Fig. 7B, C). We further analyzed the protein expression of Cyr61, LC3B, and nephrin in mouse kidney tissues by IF, and found that results were consistent with those of western blotting (Fig. 7D). Our experiments further verified that HOXA11-OS can positively regulate the expression of Cyr61 and autophagy by targeting miR-124-3p, thereby affecting the renal function of lupus mice.

## Discussion

It is well known that LN is the most common complication of SLE (Kiriakidou and Ching 2020). Although the pathogenesis of LN is well understood, research on its treatment progress is still limited, and most patients end up with chronic kidney disease or end-stage renal disease (Parikh et al. 2020). Furthermore, research on the whole molecular mechanism of SLE and LN induction is still incomplete.

lncRNAs have been shown as key participants in various biological processes and structures. HOXA11-OS is a recently discovered lncRNA (Lu et al. 2018b). Increasing studies have shown that abnormally expressed HOXA11-OS plays a key role in the occurrence and development of some diseases, and it can be used as a potential marker and therapeutic target for future disease prevention and treatment (Xue et al. 2018; Lin et al. 2020). For example, HOXA11-OS has regulatory effects on genes in oral squamous cell carcinoma, gastric cancer, prostate cancer, and various kidney diseases (Niu et al. 2020; Liu et al. 2019b; Cheng et al. 2021; Zhu et al. 2020). In the present study, we found that HOXA11-OS was highly expressed in kidney tissues, podocytes of lupus mice, and serum of lupus patients. The results of the podocyte function experiment showed that HOXA11-OS could promote the increase in autophagy factor expression and aggravate podocyte damage. In addition, knockdown of HOXA11-OS in vivo obviously reduced the expression of autophagy factors in the kidney tissue of lupus mice and alleviated kidney function damage. These results suggest that HOXA11-OS can regulate podocyte function and may play an important role in the pathogenesis of LN. There are several hypotheses on the biological functions of lncRNAs, including the ceRNA hypothesis, which has always been the focus of researchers' attention. According to this hypothesis, specific lncRNAs can upregulate the expression of miRNA target genes by reducing the damage to miRNA activity (Zhang et al. 2019; Xu et al. 2019). In the present study, we confirmed that HOXA11-OS is mainly distributed in the cytoplasm (FISH results), suggesting that HOXA11-OS can play a sponge adsorption role. The miRNA miR-124-3p has attracted much research attention. We found that the expression of miR-124-3p and HOXA11-OS were negatively correlated, and they had mutual regulation, which indicated that HOXA11-OS could act as the ceRNA of miR-124-3p.

Autophagy is considered as a double-edged sword. Excessive and insufficient autophagy both participate in the physiological processes of cells (Allen and Baehrecke 2020; Chang 2020). Autophagy has been proven to participate in the injury process of podocytes (Qi et al. 2018). The degree of autophagy is different in injured podocytes for different diseases (Xu et al. 2020). It has been shown (Yu et al. 2019) that the level of autophagy in kidney tissues and blood of LN patients is significantly upregulated and podocytes are seriously damaged. Vitamin D can protect podocytes from damage in LN patients by regulating autophagy activity. Cyr61 is a secreted protein with many biological effects (Bartkowiak et al. 2021; Fan et al. 2019). Cyr61 often shows obvious high expression during the onset of SLE. However, the specific regulatory mechanism of Cyr61 in SLE is not fully understood. It was found that the expression of Cyr61 and autophagic factors was highly expressed in the kidney tissue of lupus mice and in the serum and podocytes of lupus patients, which was similar to the expression trend of HOXA11-OS but opposite to that of miR-124-3p. Bioinformatics analysis showed that miR-124-3p could simultaneously target the 3' untranslated region of HOXA11-OS and Cyr61, which was confirmed by the double luciferase reporter gene assay. Subsequently, our in vitro experiments showed that HOXA11-OS can target miR-124-3p to regulate the expression of Cyr61 and autophagy factors through sponging, thus affecting the function of podocytes. In addition, in vivo experiments confirmed that the HOXA11-OS/miR-124-3p/Cyr61 regulatory network can play a role in LN, mainly in regulating the expression of autophagy factors and podocyte marker proteins in kidney tissues. However, the degree of autophagy was not discussed in depth in this study, and the related mechanism of clinical samples is lacking. We will complement this research in the future to further consolidate the significance and value of the present study.

## Conclusions

In conclusion, HOXA11-OS can mediate the expression of Cyr61 through miR-124-3p sponging, thus regulating the expression of podocyte autophagy factors and affecting the podocytes and kidney injury levels in lupus mice. This indicates that HOXA11-OS may be a potential molecular marker in the diagnosis and treatment of LN.

## Data Availability

All data generated or analysed during this study are included in this published article.

## Abbreviations

LN: Lupus nephritis
SLE: Systemic lupus erythematosus
HOXA11-OS: Opposite strand of homeobox A11
lncRNA: Long non-coding RNA
miRNAs: MicroRNAs
ceRNA: Competitive endogenous RNA
Cyr61: Cysteine rich 61
Beclin-1: Programmed cell death-1
LC3: Microtubule associated protein 1 light chain 3
IgG: Immunoglobulin G
AAV: Adeno-associated virus
GAPDH: Glyceraldehyde 3-phosphate dehydrogenase
CCK-8: Cell counting kit-8
PBS: Phosphate-buffered saline
DAPI: 4’,6-Diamidino-2-phenylindole
Scr: Serum creatinine
BUN: Urea nitrogen
dsDNA: Double stranded DNA
ANA: Antinuclear antibody
Sm: Smith
IF: Immunofluorescence
